# From Microorganism-Based Amperometric Biosensors towards Microbial Fuel Cells

**DOI:** 10.3390/s21072442

**Published:** 2021-04-01

**Authors:** Eivydas Andriukonis, Raimonda Celiesiute-Germaniene, Simonas Ramanavicius, Roman Viter, Arunas Ramanavicius

**Affiliations:** 1 NanoTechnas-Center of Nanotechnology and Material Science, Faculty of Chemistry and Geosciences, Vilnius University, LT-03225 Vilnius, Lithuania; eivydas.andriukonis@chgf.vu.lt (E.A.); raimonda.celiesiute@ftmc.lt (R.C.-G.); simonas.ramanavicius@ftmc.lt (S.R.); 2 Department of Physical Chemistry, Faculty of Chemistry and Geosciences, Vilnius University, LT-03225 Vilnius, Lithuania; 3 Laboratory of Nanotechnology, State Research Institute Center for Physical Sciences and Technology, LT-10257 Vilnius, Lithuania; 4 Laboratory of Bioelectrics, State Research Institute Center for Physical Sciences and Technology, LT-10257 Vilnius, Lithuania; 5 Center for Collective Use of Scientific Equipment, Sumy State University, 40018 Sumy, Ukraine; 6 Institute of Atomic Physics and Spectroscopy, University of Latvia, LV-1004 Riga, Latvia

**Keywords:** microbial biofuel cells, yeast, direct electron transfer, extracellular electron transfer, cell membrane/wall modifications, conducting polymers, enzyme-based biofuel cells, bioelectronics, microbial biosensors, whole cell-based biosensors

## Abstract

This review focuses on the overview of microbial amperometric biosensors and microbial biofuel cells (MFC) and shows how very similar principles are applied for the design of both types of these bioelectronics-based devices. Most microorganism-based amperometric biosensors show poor specificity, but this drawback can be exploited in the design of microbial biofuel cells because this enables them to consume wider range of chemical fuels. The efficiency of the charge transfer is among the most challenging and critical issues during the development of any kind of biofuel cell. In most cases, particular redox mediators and nanomaterials are applied for the facilitation of charge transfer from applied biomaterials towards biofuel cell electrodes. Some improvements in charge transfer efficiency can be achieved by the application of conducting polymers (CPs), which can be used for the immobilization of enzymes and in some particular cases even for the facilitation of charge transfer. In this review, charge transfer pathways and mechanisms, which are suitable for the design of biosensors and in biofuel cells, are discussed. Modification methods of the cell-wall/membrane by conducting polymers in order to enhance charge transfer efficiency of microorganisms, which can be potentially applied in the design of microbial biofuel cells, are outlined. The biocompatibility-related aspects of conducting polymers with microorganisms are summarized.

## 1. Introduction

A biofuel cell (BFC) is a bioelectrochemical system or device, which can produce electricity from organic materials by enzymatic catalysis or metabolic processes running in bacteria and/or other living cells. Recently this research topic has attracted increasing attention as a prospective ‘green’ technology. The most intriguing aspect of biofuel cell application is waste water treatment by simultaneous production of electricity followed by the degradation of organic-waste. BFCs can be driven by enzymes or microorganisms. Microorganism-driven biofuel cells, which are often called microbial biofuel cells (MFCs), are the most prospective because microorganisms are capable of reproducing themselves and there is no need for the purification of enzymes [1,2,3,4,5], which is otherwise a very important and costly procedure required for the development of enzymatic biofuel cells (EBFCs) [6]. Both microbial biofuel cells (MFCs) and EBFCs operate at ambient temperatures, but MFCs are cheaper and can be designed from bacteria populating sludge, soil and many other natural environments. However, the main obstacle for their wide application is a low electrical conductivity of the cell wall and cell membrane, which limits charge transfer (CT) ability and efficiency. The improvement of the electron transfer from the living cells to the anode during oxidation of organic matter is considered to be the main challenge for the enhancement of biofuel cell efficiency. Thus, researchers try to use various methods to increase the charge transfer from living cells. The extracellular electron transport towards the anode of microbial-biosensor and/or MFC is performed by internal/external electron transfer mediators, which very often are membrane-bound compounds capable of transferring electrons, or by electrode modifications that enable charge transfer to be improved [6]. The most commonly microorganisms used in direct electron transfer based biosensors and fuel cells are *Shewanella putrefaciens*, *Geobacter sulfurreducens*, *Aeromonas hydrophila, Geobacter metallic reducens* and *Rhodoferax ferrireducens*. Their catalytic activity and electron transfer route have been relatively well investigated; therefore, it is thought that physical contact between electrode and outer-membrane cytochromes or/and conductive pili of microorganisms can enable direct ‘wiring’. Catalytic activity and possible CT pathways and mechanism from eukaryotic microorganisms such as *Saccharomyces cerevisiae* is under intensive investigation [7,8,9]. It should be noted that *S. cerevisiae* recently are considered as a prospective ‘biocatalyst’ suitable for MFCs due to its broad substrate spectrum, easy and fast mass cultivation, reasonable cost and non-pathogenic material. For efficient performance of *S. cerevisiae* in MFCs, exogenous mediators are still required, because these microorganisms do not produce redox mediators indigenously [7,8,9,10,11]. It was determined that higher conversion efficiencies are observed using microorganisms capable of generating compounds that are able to act as redox mediators if compared to these microorganisms, which are not producing any redox compounds with redox mediating properties [6,12]. Therefore, MFCs, using eukaryotic yeast cells are still very rare. MFCs, based on *S. cerevisiae* extract the energy using the nicotinamide adenine dinucleotide(NADH/NAD^+^) redox cycle from anaerobic glycolysis, which takes place in the cytosol of the cell. Thus, NADH is easily accessible to a mediator molecule attached to the cell membrane. Then NADH is re-oxidized back into NAD^+^ while a redox mediator is reduced; in this way the energy extraction process in the fuel cell does not disrupt the natural metabolism of the yeast cell. In order to improve *S. cerevisiae*-based MFCs performance, various modifications are being investigated and applied, such as electrode, cell wall or membrane modifications [7,13,14,15].

The aim of this review is to overview common principles that are applied in the development of amperometric biosensors, which can be easily adapted for the design of biofuel cells.

## 2. Whole Cell-Based Biosensors

Nowadays, electrochemical (bio)sensors are of great significance in many areas [16] providing information on various analytes of interest [17]. Typically, biosensors are categorized according to their bio-recognition elements, which can be antibodies [18], receptors [19], enzymes [20], DNA [21] or living cells [22]. Whole-cell biosensors are based on microbial or other type cells acting as their bio-recognition elements. Microbial biosensors are ideally suited for the analysis of extracellular chemicals, their main advantages are: (i) ability to detect a wide variety of substrates, (ii) rather cheap and basic mass production, and (iii) easier genetic modification compared to other types of sensors and biosensors [16]. Moreover, microorganisms are able to adapt towards adverse conditions and to develop the capability to consume different chemicals over time. However, some limitations of whole-cell and microorganism-based sensors still remain, one of them being that very precise determination of target compounds is still not possible, due to the relatively poor sensitivity and selectivity. Another intrinsic limitation of microbial biosensors is their rather slow response caused by moderated diffusion of substrates into the cell through natural cell barriers such as the membrane or cell wall [17].

To overcome these drawbacks of the microbial biosensors numerous techniques are applied, such as genetic engineering in order to block undesired or induce desired metabolic pathways in the cells [23]; micro- and nano-technologies with the goals for miniaturization, high-throughput screening, enhanced sensitivity, increased selectivity and more efficient immobilization of microorganisms [17]. Attempts to overcome barriers of the cells applying various techniques include physical (freezing and thawing), chemical (dissolving within organic solvents/detergents), enzymatic (treatment by lysozyme, papain) [24] and electroporation [25] based approaches. All of these cell modification methods have some disadvantages, e.g., reduced cells viability after treatment with electric pulses [25].

During the development of whole cell biosensors, selection of the immobilization method on the electrode surface is of great importance. An ideal matrix for the cell immobilization should function at ambient temperature; enable to maintain high cell activity; prevent the cells from leakage to the buffer; allow the influx of nutrients, oxygen, and analytes as well as the removal of the resulting metabolites from the cells; and ensure electron transfer from the cell to the electrode. Entrapment into polymers of natural origin prepared from alginate/pectate, κ-carrageenan, collagen, gelatin, chitosan and agar (agarose) was performed under mild conditions with high viability of entrapped cells [22]. However, these matrices can be easily destroyed by chelating agents, which could be present in a sample, because they are formed by an ionotropic gelation in the presence of Ca^2+^ or K^+^ ions [22]. The synthetic polymers such as polyvinylalcohol, polyacrylamide, and polyurethane (PU) are more stable, but in many cases, they can induce the decrease of cell viability. The major limitation of cells entrapped within polymeric matrices is a creation of an additional diffusion barrier, which slows down the response of the biosensor [26].

A novel ‘cells-on-beads’ immobilization strategy is providing a fast and simple fabrication method suitable for the construction of viable whole-cell biosensor chip [27]. The proposed immobilization method is based on the modification of polyacrylamide porous beads with positively charged groups, which favors good *Escherichia coli* cell adsorption to the surface of gold. This amperometric biochip yielded a signal within the range of tens of nanoamperes, which was linearly dependent on the concentration of aniline [27].

A whole-cell amperometric biosensor based on ‘bioelectrochemical wire’ consisting of riboflavin-cytochrome C proteins between cells of *Shewanella oneidensis* MR-1 and working electrode was developed for riboflavin detection by using fumarate as the electron acceptor. A linear calibration curve with extremely wide riboflavin concentration (5 nM to 10 μM) was obtained, with the limit of detection (LOD) of 2.2 nM. [28]. In other study, reproducible microbial electrochemical cell-based biosensor for the determination of cyclohexane carboxylic acid in water samples was developed with cyclohexane carboxylic acid concentrations ranging from 50 to 250 mg chemical oxygen demand (COD) L^−1^. The biosensor could be used as a bioanalytical tool for monitoring naphthenic acids concentrations in oil sands process-affected water [29].

Some kinds of whole cell sensor can match requirements of enzymatic sensors due to applied genetic modifications of enzymes coding DNA sequences that are fused with DNA sequences of proteins, which are expressed on the surface of living cells [30]. Using this technology, it is possible to create cells containing enzymes of interest that are bound to the surface of the cell. This technology prevents enzyme leakage and the expressed enzymes possess high response rate, sensitivity and specificity. These systems can be based on various types of cell, including bacteria, fungi or yeast [31]. The main drawback of such systems that not all DNA sequences of enzymes can be genetically fused with that of native cells’ extracellular surface proteins and they do not always retain normal catalytic activity because the fusion of proteins can disrupt their normal folding procedure.

## 3. Electrode Modifications for the Improvement of Charge Transfer in Biosensors and Microbial Biofuel Cells

**Figure 1:**
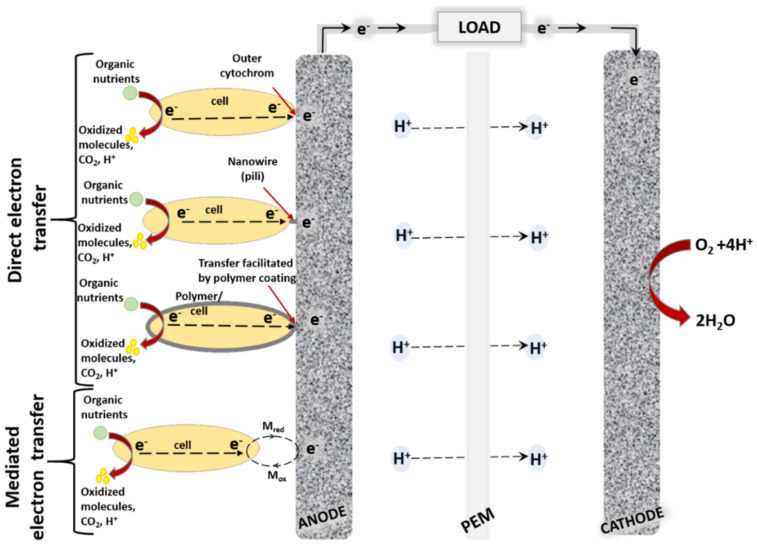
General scheme of microbial biofuel cell with direct and mediated electron transfer.

The characteristics of anode material play a critical role in amperometric biosensors and MFC, because the nature of material is among the key factors, which significantly affects charge transfer efficiency from biomaterials, and it is one of the major reasons causing the rather low efficiency of some MFC prototypes. In order to maintain the viability of immobilized microorganisms the most efficient anode material should be biocompatible with these microbes, should have a high surface area for biomaterial to adhere to, and should be susceptible to robust microbial cell attachment. At the same time, materials used for the modification of anode should facilitate electron transfer, which is a limiting factor for the sensitivity of amperometric biosensor or the efficiency of MFCs. The adhesion of microorganisms on the electrode surface plays one of a key roles in the electron transfer process and generated power density [12]. The most popular MFC design is based on two-compartment cell with microorganisms enclosed in an anode compartment and/or sometimes even in a cathode compartment. Between compartments, the ion uptake is required and, therefore, components should be divided by semipermeable membrane, which enables the exchange of some ions. The extracellular charge transfer from microorganisms towards the anode is facilitated by internal or external electron mediators or membrane-bound compounds, which are capable of electron transfer [6] (Figure 1).

Commercially available state-of-the-art membranes suitable for MFC are often modified by perfluorosulfonic acid (Nafion) layer, which exhibit a high proton conductivity and chemical stability and are the most popular for the application in MFCs. However, despite numerous advantages Nafion membranes significantly increase the cost of the system in comparison to those based on other types of membranes or membrane-less MFCs. In addition, Nafion-based membranes become fouled during the action of MFCs and, therefore, researchers are attempting to substitute them by other material-based membranes [32,33].

The most commonly utilized anode material in the MFCs are carbon-based materials, because they have high conductivity, they are chemically stable, and relatively cheap. Nevertheless, carbon materials are intrinsically hydrophobic, which is unfavorable for robust attachment of living cells, resulting in non-efficient charge transfer. To resolve this problem, various chemical and physical modifications were involved to enhance the properties of carbon-based materials. However, the effect of modifications makes it important to compromise between low cost and biocompatibility, while supporting strong attachment of microbes and facilitating charge transfer towards electrodes, which are extremely important for optimal MFC performance. In addition, modifications should also provide good electrical conductivity with low resistance, large surface area, anti-corrosion and anti-degradation properties, suitable mechanical strength and toughness [34]. The most common modifications of electrodes are based on the functionalization with carbon nanomaterials [35,36,37,38], metal oxide nanoparticles or their nanocomposites, conducting polymers or the formation of various nanocomposites based on conducting polymers [34,39].

Carbonaceous nanomaterials provide a large surface area and extraordinary electron transfer properties. Carbon felt (CF) is commonly used as a low-cost anode material, which is very suitable for MFCs design due to a good electronic conduction, high surface area and porosity enabling sufficient mechanical stability to be provided and, therefore, carbon felt seems to be a favorable material for biofilm formation [40,41]. However, highly hydrophobic nature of carbon felt makes their application more complicated in aqueous electrolyte medium and for the population of this structure with living cells. To overcome this drawback, many modification methods have been developed and used resulting into a new and benefiting composites. Chemical modifications change the hydrophobic surface of bare felts to hydrophilic. For example, polyethylenimine has a high positive charge density and can interact through its abundant amine groups with the carbon-based materials [42]. This material is commonly used for the modification of carbon felt anodes employed in MFCs [43,44]. Carbon nanotubes (CNTs) have shown controversial characteristics towards living cells [45] thus, the anode modifications of the MFCs often go together with biocompatible polymers, such as polyurethane (PU) [20], or polyaniline (PANI) [46,47,48].

The influence of polyurethane on biocompatibility and adhesion-activity of carbon anode with the composite of polyurethane/carbon nanotubes composite was investigated [14]. Polyurethane is known as a biocompatible material [46], thus the composites with CNTs have less effect on the biological activity of living cells used in amperometric biosensors and MFCs. The porous polyurethane sponge provided a vast surface area for physical CNTs adsorption as well as for cell biofilm formation. Daily substitution of analyte allowed the MFC to operate at 70% of maximum power (100 mW m^−2^) for long periods. Thus, this MFC has a prospect as a low-cost energy generating device [14]. To separate anode and cathode chambers, the aforementioned ion exchange separator based on (polystyrene-polyvinyl alcohol)/(phosphoric acid) membrane was applied in the design of this MFC. This membrane proved to enhance proton conductivity in dehydrated as well as hydrated states, with an estimated ion transfer number in order of 0.97 as well as had an antifouling effect [14] unlike the usually used Nafion membranes [32] (Table 1). In order to avoid the application of ion exchange separators, membrane-less BFCs are designed [49]. Stability and compatibility of membraneless MFC based on yeast/CNT catalyst were investigated and catalytic activity of this MFC was evaluated [49]. In this study, carbon nanotubes and poly(ethylenimine) were selected as a supporting material for the immobilization of the yeast cells based on hydrophobic interactions between CNTs and yeast cells followed by the cross-linking technique. It was proved that yeast/CNTs induced excellent catalytic activities and MFC performance (344 mW m^−2^) with MFC stability of 86% from its initial value after eight days (Table 1) [49].

Strategy for anode modification with multiple bilayers of CNTs/PANI decorated indium tin oxide (ITO) electrode with a nanoporous network exhibited superior biocatalytic properties with a maximal current density of 6.98 μA cm^−2^ and maximum power of 34.51 mW m^−2^ (Table 1) [47]. The layers of PANI were graft-polymerized via covalent bonding while the CNTs were assembled applying layer-by-layer deposition technique, which ensured low CT resistance. The modified electrode had more than seven times higher maximum power density in comparison to that registered by the unmodified ITO electrode. This result indicates that CNTs/PANI conductive films greatly increases the area of electrode surface and the number of sites that are involved in charge transfer from bacteria. In addition, the toxicity of the CNTs toward the *Shewanella loihica* cells was minimized by modification with biocompatible polymer-PANI [47]. The genus of *Shewanella* is known as metal-reducing bacteria and, therefore this type of MCFs could be applied for the control of chromium ion-based pollution as well as the production of chromium-based nanoparticles [50] that can be harvested from formed sediment.

Facilitation of charge transfer from biological objects by redox polymers is discussed in the work of Coman et al. [51]. In this study researchers explored the applicability of flexible osmium polymers (poly(1-vinylimidazole)_12_-[Os-(4,4′-dimethyl-2,2′-bipyridyl)_2_Cl_2_]^(+/2+)^ (I) and poly(vinylpyridine)-[Os-(N,N′-methylated-2,2′-biimidazole)_3_]^(2+/3+)^ as a matrix material and for electron transfer between the cells and the electrode. *Bacillus subtilis* were suspended in dried polymer, thus wiring them through the polymer matrix to the electrode (Table 1). Wired bacteria managed to reach 5 µA cm^−2^ current density supplied with 0.3 mM succinate using an aldrithiol-modified gold electrode. A stability test showed slightly decreased current response over time and they reached approximately 73% of the initial response after 6 h.

New strategies for the modification of the anode of a MFC with alginate film were proposed by another group of researchers [52]. Neutral red (NR) was used as a mediator in this electrochemical system. The anode was modified with yeast by drop-coating method, and yeast film was formed by physical adsorption using immersion of carbon felt into yeast-alginate suspension. To determine anode performance, electrochemical characterization was performed, which proved that the deposit of an alginate film entrapping yeast cells is an efficient way to promote glucose oxidation and to transfer electron. Performance of the MFC were evaluated in terms of maximum power generation (0.326 A m^−2^) at the load of 1 kΩ. Such MFC had operational stability for a period of 44 days. The current of MFC partially decreased due to the reduced activity and viability of immobilized cells. It was stated that another approach when yeast cells were entrapped within alginate–NR microbeads, then current was limited by obstruction of the NR reduced form, which was exiting the matrix gel layer towards the electrode surface [52].

To improve carbon-based anode materials, the performance of conductivity, electrocatalytic activity, various noble metal and metal-based materials are utilized to modify carbon materials. Nanoparticles act as the bridge for electron transfer between bacteria and anode. In contrary to carbon nanomaterials, metal nanoparticles are not very widely applied because of their corrosiveness [53] and cytotoxicity [54]. However, gold nanoparticles (AuNPs) and their composites are considered as suitable for electrode modification in bioelectrochemical devices due to their wide array of beneficial properties, such as biocompatibility, high surface-to-volume ratio, and enhanced conductivity [55].

Biogenic golden nanoparticles (BioAu) and the composite with multiwalled CNTSs (BioAu/MWCNT) was used for the modification of carbon cloth anodes for MFC construction [56]. Biologically produced AuNPs are known for their low toxicity, high purity and biocompatibility thus allowing the aforementioned general drawbacks of metal-based nanoparticles to be minimized. The biofilm was constituted mostly from the classes of *Gammaproteobacteria* and *Negativicutes*, which increased after anode modification. The MFCs with the bioAu/MWCNT electrode had the highest power density of 178.34 ± 4.79 mW m^−2^ and operation time of shorter than 7 days (Table 1), which in the term of power density was 56% higher and in the term of operation time was 142% shorter compared with these characteristics of unmodified control electrode showing the strong affinity between the electrode surface and materials used for the modification [56]. Authors in this study attempted to investigate the electrochemical MFC performance and microbial community behavior affected by novel anode modification based on biogenic Au nanoparticles.

Duarte et al. developed and characterized golden nanostructures grown on a polyethyleneimine functionalized carbon felt substrate as an anode material of MFC. The gold nanoparticles growth process utilized surface-bound seeds. The developed widespread gold nano-flower structures had an irregular shape, which proved to be very beneficial for the physical adhesion and inhabitancy of the yeast cells. The maximum power density achieved was 2771 ± 569 mW m^−2^ for the polyethyleneimine-modified carbon felt with gold nanoparticles prepared with 715 μM 4-mercaptobenzoic acid after only 30 min after preparation (Table 1). The higher power density, which was achieved by this approach was affected by AuNPs that bridge the external cellular wall of the yeast cells and the surface of carbon fibers. The contribution of AuNPs for direct electron transfer enabled ‘local harvesting of more electrons’ and the accumulation of charge on the CF electrode [44]. The same authors later developed metal composite nanostructures, namely manganese oxide-decorated iron oxide nanoflowers on the same anode material- polyethylenimine functionalized carbon felt. A very efficient electrochemical interface between the yeast biofilm, metallic nanoflowers, and carbon felt fibers was constructed, further enhanced by the inclusion of the anionic surfactant mediator, sodium dodecylbenzenesulfonate (SDBS). Biofilm formation was performed by physical attachment of yeast cells on the anode material. When SDBS was used, the nanostructures stayed firmly attached to the carbon felt fibers due to the additional anionic interactions strengthening the entrapment bonds of the polyethylenimine-coated fibers. This is supported by the increased surface coverage activity of the samples containing SDBS. Extracellular polysaccharides of the yeasts cell wall directly influenced the direct electron transfer properties of the yeast and, thus, the functionality of the MFC. The external nutrient-enriched and highly electrochemically responsive extracellular polysaccharide matrix reached out around the individual FeMnNPs on the electrode surface and created a strong electrochemical bridging effect between the yeast cells and nanostructures. The best power density of 5.83 ± 0.61 W m^-2^ was achieved (Table 1) [43].

In the work of Christwardana et al. [57], the quorum-sensing molecules, which are employed by microorganisms as a major means of communication and biofilm formation [58] were used for MFC anode functionalization to ascertain the suitable surface for microbial attachment, to enhance the biofilm formation, activity, and conductivity for the electron transfer and electron–electrode interaction in MFCs [59]. This scheme of the anode functionalization proved to be rather efficient, as the maximum power density of the MFC were 159.46 ± 10.68 mW m^−2^ and 156.57 ± 5.84 mW m^−2^, using different quorum sensing molecules phenylethanol and tryptophol, respectively. The third quorum sensing molecule, tyrosol was slightly less effective (Table 1). As seen from the Table 1, the constructed MFC did not have well-expressed maximum power density, however the idea of using quorum-sensing molecules was promising because of the possibility to speed natural formation of the biofilm. The presence of extracellular polysaccharide in a biofilm did enhance the direct electron transfer between yeast cells and working electrode surface. The improvements by using microbial consortiums, electrode surface modifications and other methods for the sophistications that could be applied are suggested by authors [59].

Another group of authors presented a biofilm of fungi *Scedosporium dehoogii*, which was used for the modification of the CF anode in a MFC for potential bioremediation of a toxic pharmaceutical compound para-aminophenol from wastewater [60,61]. *S. dehoogii* is a fungus able to use aromatic hydrocarbons as an energy source; thus, it can be simultaneously applied for two purposes: bioremediation of waste water from toxic contaminants as well as for energy production. The cathode of MFC was a CF electrochemically modified by electrodeposition of a poly-Ni (II) tetrasulphonated phthalocyanine (poly-NiTSPc) film, which replaced the classical Pt/Air cathode by the modification of carbon felt surface with poly-NiTSPc. This kind of modification aimed to increase the intensity of O_2_ reduction, leading to an increased performance of the MFC [60,61]. Waste waters generally contain low concentrations of pharmaceutical compounds and thus cannot allow a MFC to attain a high-power density. Therefore, the additional source of electron donorcellulose-based fuel was evaluated. It showed a maximum power density of 16 mW m^−2^ for a time of 200 h (Table 1).

As the conductance of the conductive polymers themselves is relatively low, other materials, such as carbon nanotubes, are researched for the modifications of anode surface to promote effective direct electron transfer [7]. It was determined that at lowest explored concentration (2 μg mL^-1^) of MWCNTs and at rather short-lasting exposure of MWCNTs do not significantly affect the viability and other properties of yeast cells. The results obtained from electrochemical characterization showed MWCNTs being a very good candidate for the development of MFCs, as the application of MWCNTs in the anode of the biofuel cell increased generated power by 69 times (113 nW cm^-2^) (Table 1). Therefore, authors suggest, that MWCNTs can be applied for the modification of the electrode in order to improve electrical CT through the yeast cell membrane and/or cell wall. Evaluations based on fluorescence microscopy and cell count revealed that the viability of the cells was not affected by MWCNTs when the concentration was of 2 µg mL^-1^ [7].

As seen from the Table 1, the highest maximum power density was achieved using CF anodes, which were decorated with golden nanoparticles [44] or manganese oxide decorated iron oxide nano-flowers [43] and then modified by *S. cerevisiae,* and in both cases the extracellular polysaccharide matrix of *S. cerevisiae* interacted with NPs, which were deposited on the electrode surface, and created a strong electrochemical bridging between the yeast cells and applied nanostructures. A very advantageous surface of the carbon felt anodes providing a high surface area as well as good physical surface characteristics for microbial attachment and direct electron transfer was achieved in both cases [43,44]. The Nafion membranes were used in most cases for the separation of ion exchange. The different strategy was developed by de Oliveira et al. [14], which enabled to achieve more stable membrane. Biomaterials such as algae [52] and quantum sensing molecules [57] were employed for better microorganism entrapment to the electrode surface, and in the alginate case the extraordinary stability for 44 days was achieved most likely owing to biocompatible environment provided by algae preventing the cells from leakage to the buffer.

The applicability of the constructed MFCs is rarely discussed in the reviewed papers due to them still being in an early stage of development. Thus improvements for generated power density, stability and other properties still needed. The *S. dehoogii* based MFCs showed organic micropollutant para-aminophenol as an efficient model fuel for this MFC, which could be applied for the bioremediation of this toxic pharmaceutical compound. Moreover, the aforementioned devices were stable for more than 8 days [60,61]. *S. loihica* is known to reduce metals, thus the MFC based on this microorganism [47] could be applied as an environmentally friendly and nontoxic approach for the production of chromium nanoparticles as well as for the remediation of chromium and its contamination [50]. MFCs based on classes of *Gammaproteobacteria* and *Negativicutes* were applied for bioremediation and energy generation from sludge [56]. In many cases the main focus of the researches were on the electrode surface modifications with various materials as the guide for better strategy in order to develop the devices with better performance [7,14,43,44,49,51,57] rather than a MFC ready for applications. In many of the aforementioned cases *S. Cerevisiae* was used as a model microorganism.

## 4. Electrochemically Deposited Conducting Polymers for Better Biocompatibility of MFCs

**Figure 2:**
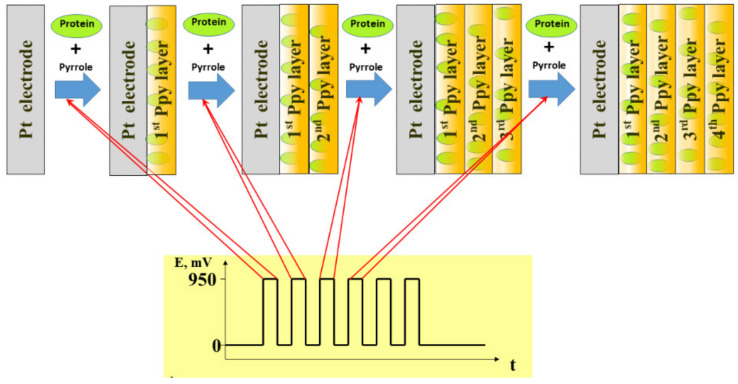
Electrochemical deposition of conducting polymer–polypyrrole and entrapment of proteins within formed Ppy layer, while potential pulses are applied. Adapted from [62].

Electrochemical deposition of conducting polymers is rather simple method for the modification of electrode surface. This method is frequently applied during the development of bioelectronic-based devices. The number of electrical characteristics (such as electrochemical technique, working electrode potentials that are required for the initiation of monomer polymerization, etc.) can be varied and easily adapted during the formation conducting polymer layer with required physicochemical performance [62,63]. Therefore, the physical characteristics of formed layers such as layer thickness, density, ion-permeability can be adjusted by the adaptation of optimal electrochemical conditions required for polymerization reaction. Moreover, many chemical parameters including solvents, polymerizable monomers, polymerization bulk composition, and pH are strongly affecting characteristics of formed CP layers. During the course of electrochemical polymerization some biologically active materials such as proteins [64,65,66,67], DNA [21,68] and even living cells and bacteria can be entrapped within the conducting polymer-based layer when they are added into a polymerization solution. The scheme of electrochemical deposition of Ppy layers with entrapped protein molecules [69] is presented in Figure 2.

**Figure 3:**
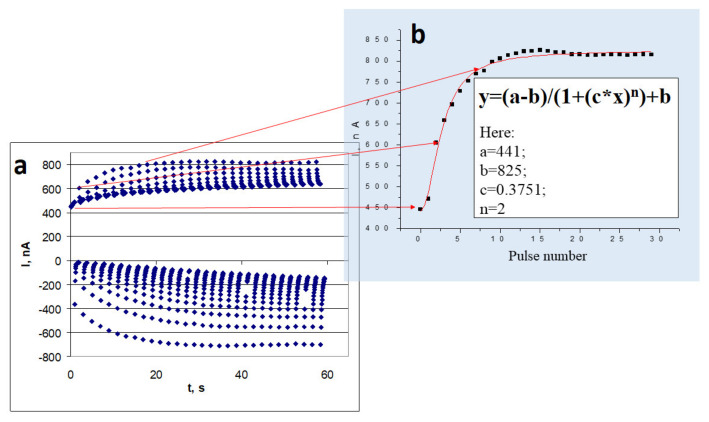
(**a**) Chrono-amperogram, which was registered during electrodeposition of Ppy, using pulsed-potential-based mode. (**b**) Dependence of anodic peaks, which are presented in Figure 3a, on the pulse number during electrochemical deposition. Adapted according to data presented in [69].

The application of suitable electrodeposition conditions enables the efficiently of conducting polymer layers to be adapted in the design of bioelectronics-based devices [70,71,72]. The diffusion rate of the nutrients, which are acting as microbial biofuel is a very important factor for the performance of MFCs. Therefore, sometimes it is reasonable to change the porosity of the formed CP-based layer by the incorporation of some organic-based ‘spacers’, which are interlinking different polymeric chains [73]. The conductivity of CPs-based layers is also very important for the efficiency of MFCs. Here the achievements of some research groups in the evaluation of synthesis parameters on electrical conductivity of electrochemically deposited Ppy films [74] and PANI-based layers [75] (Figure 3) [69] as well as mathematical model, which was derived by our team [76] can be exploited.

**Figure 4:**
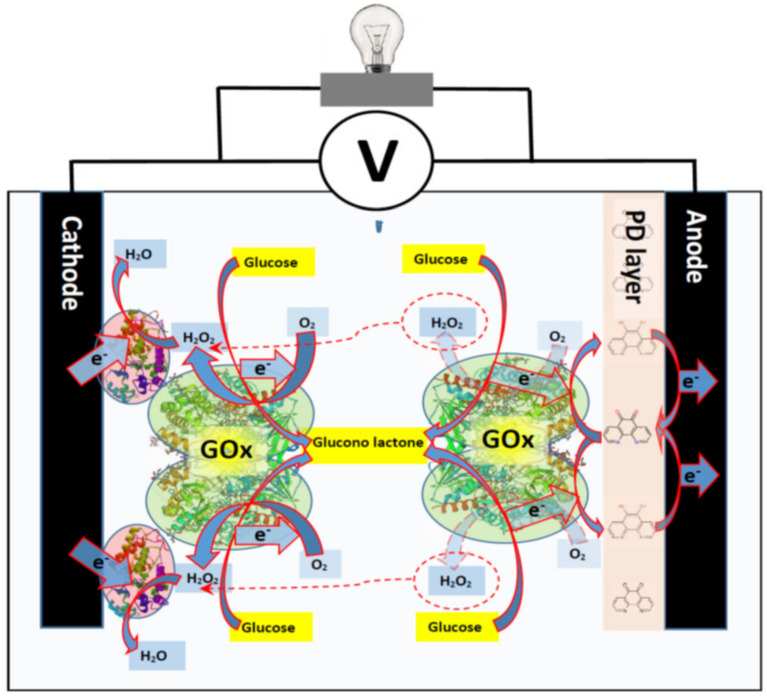
Enzymatic biofuel cell powered by glucose, cathode of this biofuel cell is based on co-immobilized horseradish peroxidase (HRP) and glucose oxidase (GOx) and anode is based on immobilized glucose oxidase. On anode electrons from GOx are transferred to the electrode via redox mediator power density (PD) and in cathode hydrogen peroxide created during enzymatic reaction of GOx is consumed by HRP and electrons from cathode are transferred directly to HRP.

The concentration and viability of entrapped cells are also very important for the performance of amperometric biosensors and biofuel cells. Therefore, the compatibility of conducting polymers with microorganisms play a very important role for the performance and application of these bioelectronics-based devices. Such bioelectronics-based devices find new application areas in biomedicine including that in biomedical implants [77], which need suitable long-lasting power sources [78]. In this context, the most suitable power sources for such devices could be enzymatic or catalytic biofuel cells, because they can use unlimited resource of biofuel (e.g., glucose) from our organisms (Figure 4).

Despite diverging opinions regarding the applicability of biofuel cells [79,80], advantages of them were demonstrated by the implantation of BFCs into plants [81,82], animals such as rabbits [83], rats [84,85,86,87], clams [88], insects [89], snails [90], and even the human body [91,92]. Therefore, the demand for BFCs is constantly increasing, but many specific challenges are arising in this area of technology [93]. Biofuel cells can be implanted together with powered device, but biocompatibility of implanted BFC is the most important issue, because the electrodes of BFCs differently from many other parts of the device should be in direct contact with body liquids, otherwise the BFC will not operate. Hence, a very important issue is the biocompatibility of materials that are in direct contact with body tissues. If the materials that are contacting with body liquids [94] lacks biocompatibility then allergic and inflammatory reactions can be induced [95,96]. Therefore, the selection of proper materials capable to retain sufficient functionality of immobilized biomaterial is the most important for the development of bioelectronics-based devices [97,98]. CPs are among such materials that can effectively cover surfaces of electrodes with layers modified by biological objects, therefore, numerous research works evaluated some aspects of CPs’ biocompatibility with proteins [1], DNA and stem cells [99,100]. Despite these studies, just limited number of researches have been dedicated for the investigation of CPs’ influence on immune system of laboratory animals [101]. Our research team has conducted research in this direction by the evaluation of effect towards more advanced ‘biological systems’ such as living stem cells [99,100] and on immune system of laboratory mice [101]. It was demonstrated that Ppy only slightly affects the immune system of these laboratory animals [101]; some influence of Ppy on bone marrow-derived stem cells was determined when higher concentration of Ppy nanoparticles was applied, and by contrast, if a low concentration of polypyrrole nanoparticles was used, then the toxicity towards mouse hepatoma (MH-22A), human T lymphocyte Jurkat, and primary mouse embryonic fibroblast cells was not observed at all [99]. Hence, these investigations revealed that Ppy is biocompatible with evaluated cell-lines [99,100] and immune system of laboratory animals [101]. In addition to Ppy, some aspects of biocompatibility of another conducting polymer–polyaniline (PANI) were also investigated [48]. It is interesting that electric field based stimulation induces the differentiation of nerve cells, which were deposited on a hetero-structure based on polypyrrole/poly (2‑methoxy-5 aniline sulfonic acid) [102]. If necessary, the biocompatibility of CP-based structures can be advanced by mixing them with biocompatible polymer–chitosan [103,104,105] or forming water-rich hydrogel-based structures [106,107]. CP/gel-based structures can be used as scaffolds for growing cells that are used for tissue engineering [108,109], and transplantation [110] or in other biomedical applications [111,112]. Superior biocompatibility of Ppy with biomaterials enables the application of Ppy in the design of various BFCs that potentially can be integrated and implanted together with other biomedical devices.

## 5. Modifications of Microorganisms to Improve MFCs Performance

Different microorganisms [113,114] and living cells of mammalians, namely lymphocytes [115] and erythrocytes [116] can be used in the development of BFCs, but the electron transfer from microorganisms to the electrode is very rarely observed, therefore, it is a significant challenge when these bioelectronics devices are designed. The involvement of microorganism metabolic processes in the polymerization processes of conducting polymers is a very attractive strategy, which is useful during the modification of microorganisms [10,11,117,118], because conducting polymers can improve electron transfer efficiency of microorganisms, which were modified in such way. In our previous works it has been shown that stem cells [99,100] and some microorganisms [10,119] after the modification by CPs can retain viability and still can perform metabolic processes, which can be applied in the generation of electrical current by biofuel cells. The formation of CPs inside of microorganisms seems very innovative and the application of such modified microorganisms in biofuel cells and biosensors is more progressive, because living cells remain biocatalytically active for a longer time [120] when compared with the activity of concocted polymer modified enzymes [121,122]. For this reason, several types of cells [15], bacteria [123] fungi [117] and yeast [11] were engaged in the formation of various polymers, including conducting polymers.

An alternative way to improve MFCs’ performance is to target microorganism charge transfer via cell wall and/or membrane. Until now the preferred method has been based on the application of redox compounds capable to assist charge transfer, these redox compounds can be suspended or dissolved in cell suspension and not anchored to electrodes. These molecules are commonly known as redox mediators and are well investigated in numerous researches [7,8,9,122,124]. Redox mediators can be assigned into few groups: (i) hydrophilic and (ii) lipophilic mediators, (iii) nanoparticles of various origin [124,125], and (iv) conducting and/or redox polymer-based matrices [119,126,127]. If redox mediators are properly applied in the design of biofuel cells, they are able ‘to wire cells with electrodes’ and are providing more efficient at charge transfer from cell cytoplasm. Hydrophilic mediators usually enhances MFC performance by interaction with cell trans-plasma membrane redox system [128]. This interaction is thought to occur between mediator and redox enzymes that are located in the cytoplasm. The most common examples are various membrane bound cytochromes [129]. Cytochromes typically possess a redox active center, a functional group, a co-enzyme or a whole cascade of them [130]. Usually, it is thought that hydrophilic enzymes cannot pass through the membrane. Hence, here lipophilic mediators play their role in the charge transfer trough cell membrane. These redox mediators are capable of dissolving into plasma membrane and thus can easily transport charge from cell internals to the cell membrane’s outer surface. Lipophilic mediators charge transfer/migration perform via redox capable functional groups. Nonetheless, the most commonly lipophilic mediators are used in tandem with hydrophilic ones. Thus, significant improvements of charge transfer are achieved [128].

**Figure 5:**
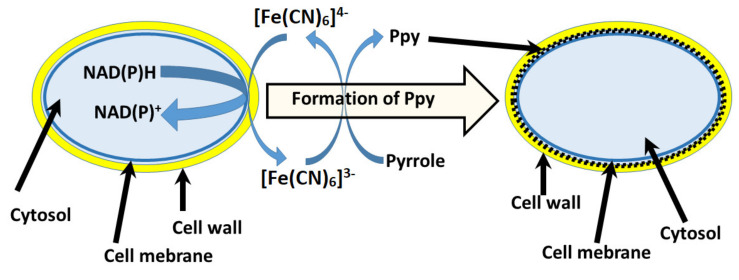
Schematic representation of Ppy synthesis in cell wall of yeast [10]; Redox enzymes that are located in plasma membrane are oxidizing [Fe(CN)6]^4−^ into [Fe(CN)6]^3−^ that is inducing polymerization reaction of pyrrole [135].

On the other hand, various types, shapes or origin nanomaterials are often implied in MFC’s. The most often carbon based nanomaterials or metallic nanoparticles play major role for improving charge transfer from redox enzymes [7,131,132]. Modification involves preparation of suspension [133] or either attachment of nanoparticles to cell surface in order to create electrical pathway from cells to electrodes. By contrast with electrode modifications, the cells are conjugated with nanoparticles rather than anchored to electrodes itself [134]. The aforementioned cell modification methodologies can be considered ‘traditional’. Recently, self-encapsulation of cells with polymers has emerged. By contrast with other methods, polymer matrices can be prepared in situ with cell culture or sometimes even produced by metabolic/chemical processes that are running inside of cells. Currently, the application of Ppy rises in the field [118]. Very first study, which is reporting Ppy bio-assisted polymer synthesis, to our best knowledge was published in 2016 by our research group [123]. It was reported that bacteria *Streptomyces* spp. are able to catalyze spherical Ppy particles formation without any additional chemicals, because bacteria *Streptomyces* spp. are able to secrete redox-enzymes (e.g., phenol-oxidase) to extracellular media. These enzymes are capable to initiate polymerization of different phenol-based monomers such as Ppy. Thus, it was demonstrated that phenol-oxidases can be exploited for the synthesis of polypyrrole. After 6 days of growing *Streptomyces* spp, bacteria were able to create favorable conditions for the formation of hollow Ppy microspheres. Hollow microspheres where of 10–20 µm in size. Researchers discussed that particle shape were influenced by organic compounds present in growth medium. Later on, it was reported that it is possible to coat yeast *S. cerevisiae* cells with Ppy [10,135]. In this case, we have used yeast cells to cycle redox mediator [Fe(CN)_6_]^3−^/[Fe(CN)_6_]^3−^ and thus to perform Ppy synthesis in situ, in a controlled manner (Figure 5). We have suggested some insight into the possible formation mechanism and documented cell viability and Ppy effects on cells and possible locations of the formed polymer. In further studies we have evaluated mechanical properties of Ppy coated cells. Additionally applying isotope ratio mass spectroscopy and non-radioactive isotopic monomer label, we were able to evaluate amounts of Ppy, which forms intercalating matrix in cell wall [11,135]. After the modification by Ppy, the microorganism remained viable, which is very important for the development of long lasting microbial biofuel cells. It was also observed that formed Ppy structures were inter-growing trough yeast cell-walls and were strongly affecting physical and chemical properties of modified cells, because cell walls became resistant to yeast wall lysis enzymes [11]. At the same time it was shown that using iron nitride iron (III) nitrate nonahydrate it was possible to form similar Ppy structures based on various bacteria: *Shewanella oneidensis* MR‑1, *Escherichia coli*, *Ochrobacterium anthropic* or *Streptococcus thermophiles* [136]. During the preparation, bacterial cells were soaked with iron (III) nitrite nonahydrate, which was located in cell outer layers and further upon introduction of pyrrole performed polymerization of Ppy. It was reported that bacterial cells remain viable and coating procedure does not affect proliferation. Considering the electrical properties, the modification with conducting polymer–Ppy–has improved power density by 14.1 times compared to that of unmodified *S. oneidensis* (147.9 μW cm^−2^). Similar self-encapsulation of microorganisms with the Ppy technique also were performed and analyzed for MFC application using *Aspergillus niger* and *Rhizoctania sp.* [117,119,137]. For MFC evaluation, scanning electrochemical microscopy (SECM) was applied [118]. The study reported that during electrochemical probing over immobilized modified cell culture current output (I_max_ = 0.86 nA) was up 3 times greater when compared to that (I_max_ = 0.30 nA) of control group [118]. Results were determined by the registration of surface approach curves. These experiments also showed how charge transfer efficiency, which is crucial for current generation in MFC, depends on several factors: (i) the distance between ultra-micro electrode of SECM and cells and (ii) surface modification of the microorganism. Nominal current output when the ultra-micro electrode was 20 µm apart from test sample was 0.47 nA, which was 1.5 times greater than that of the control sample. White-rot fungal strains belonging to *Trametes spp*. ware also modified with Ppy [118]. Researchers pointed out that Ppy formation in fungal hyphae were achieved using laccase enzyme, which is produced and secreted to growth medium by *Trametes* spp. fungi. Polymerization of pyrrole in crude enzyme extract and with cell culture in growth medium was observed. Bio-assisted polymer synthesis at that time was very innovative [138] and, according to our best knowledge, it was one of the first studies that facilitated practical application of enzyme-assisted formation of conducting polymers [70,138,139,140,141], and later led towards polymer-based coating formation in cell culture [10,11,117,118]. Thus, it was demonstrated that cells modified with conducting polymer have advanced electron transfer ability, which enables to use these microorganisms in microbial biofuel cells (MFCs) [119] and biosensors [137,142].

The most interesting application of living cell-induced Ppy formation was demonstrated by the modification/coating of mammalian cells by Ppy [143]. Researchers have applied the synthesis method, which was proposed in our earlier research [10] (Figure 5), and they were able to produce Ppy using suspension of the leukemia cell line, K562 cells. They determined that previous suggested pyrolle polymerization mechanism could be driven by cell exudate molecules, not by plasma membrane oxido-reductase systems differently from the mechanism, which is presented in Figure 5. During the polymerization of pyrrole they observed cancer cell death, which provides another application of this pyrrole polymerization mechanism as ‘reverse pro drug’ systems, meaning that cytotoxic pyrrole after cell death tends to polymerize, which yields biocompatible conducting polymer–polypyrrole [101] that is black-colored and, therefore, can also be used as optical indicator of dead cells. Following this, it was reported that implementing FeCl_3_ compound with metal reduction-capable bacteria: *Cupriavidus metallidurans*, *Escherichia coli* and *Clostridium sporogenes*, could initiate atom transfer radical polymerization (ATRP) of various monomers (poly (ethylene glycol methyl ether methacrylate); hydroxyethyl methacrylate; N-hydroxyethyl acrylamide; 2-acrylamido-2-methyl-1-propanesulfonic sodium; 2-(methacryloyloxy) ethyl dimethyl-(3- sulfopropyl) ammonium hydroxide [144]. Researchers suggested interesting approach for designing cell-assisted polymerization. Synthesis not capable Fe (III) compound is reduced into Fe (II) in controlled way and thus initiates the polymerization of monomers that are not toxic to cells, which are engaged in redox processes of Fe (II)/Fe (III). After ATRP polymerization, cells still maintain high viability.

In addition to Ppy some other polymers are also used for cell modification in order to increase their performance in designed MFC. In similar fashion *S. xiamenensis* were coated by polydopamine (PDA) [145]. Selected bacteria can adhere to PDA during their formation via oxidative polymerization in aerobic, slightly alkali (pH 8) conditions. Researchers report PDA modified bacteria *S. xiamenensis* cells were able to generate 452.8 mW m^−2^ power density, which was 6.1 times greater than that for electrodes based on non-modified cells (74.7 mW m^−2^). Conducting PDA additives were formed within 3 h, which is rather fast, and it seems also that bacteria modification just barely influence cell viability, which dropped only by 2–3%. Rather, popular bacteria for MFC design is *Shewanella oneidensis* MR-1, which was also coated with PDA. In study [146], Yu et.al. report that it is possible to use cell-assisted synthesis for the formation of conducting PDA or using the same bacteria exploit bio mineralization of FeS nanoparticles. Results show that different interfaces wire up cells at different levels and thus their electric/electrochemical properties are different. They also showed that by polysulfide reductase mineralized FeS nanoparticles interface increase the efficiency of MFC anodes up to 3.2 W m^−2^, which was 14.5 times higher than that of anodes modified by native *S. oneidensis* cells (0.2 W m^−2^), while for PDA-coated anodes current density was of ~0.6 W m^−2^.

An alternative method was demonstrated in some other research [147,148]. Researchers managed to feed or internalize pre-synthesized carbon dots (CD, carbon nanoparticles) into *S. oneidensis and Shewanella xiamenensis*, accordingly [147,148]. Both studies showed remarkable effects of carbon dots, because they are highly biocompatible. Also, carbon dots are able to enhance metabolic activity, because internal adenosine triphosphate (ATP) levels were significantly elevated. It was hypothesized that facilitated metabolism could also produce unwanted reactive oxygen species, but it was not the case. Also, carbon dots form photoactive particles, which promote lactate consumption ogether with current generation upon illumination. With *Shewanella oneidensis* MR-1 maximum current density achieved was 1.23 A m^−2^ while control was 0.19 A m^−2^. Meanwhile, the maximum power density of the MFC with carbon dots were 0.491 W m^−2^, which was by 6.46 folds higher in comparison to that of the control based with not modified same microorganisms (0.076 W m^−2^). *Shewanella xiamenensis* upon illumination conditions and sole lactate carbon source were able to reach the density of 329.4 µA cm^−2^, which was by 4.8 times higher than that of the control electrode (68.1 µA cm^−2^). Osmium redox polymers are also applied in the development of MFC [51,149,150,151,152]. In study [149], cells were trapped and wired to electrode surface via [Osmium (2,2′-bipyridine) (poly-vinylimidazole)_10_Cl] Cl. The pre-synthesized polymer was used as co-mediator and as a conductive binding matrix for *Gluconobacter oxydans* bacterial cells. Electrodes were designed via the drop coating methodology onto glassy carbon paste electrode. Researchers achieved maximum charge density of 15.079 mA cm^−2^ and open circuit potential value of 176 mV.

**Figure 6:**
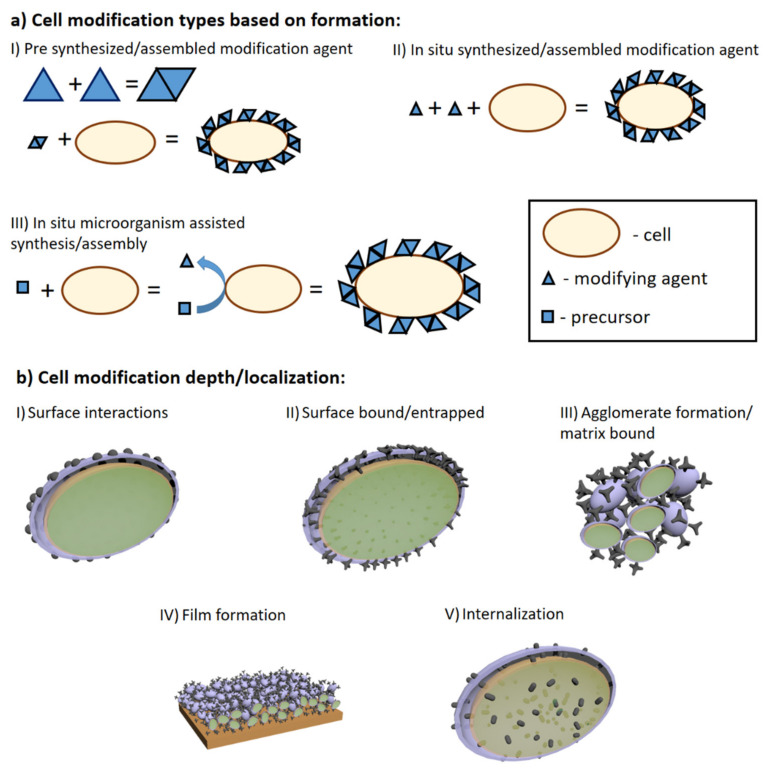
(**a**) Schematic representation of cell modification by agent formation principle. Cells can by modified using pre-synthesized compounds (I), assembled/ synthesized in situ with living cells are present (II) and in situ when cell assists/catalyzes synthesis assembly of modification agent; (**b**) schematic representation of modifying agent localization in MFC applications: (I) surface interactions as adsorption, electrostatic interactions etc.; (II) modifying agent is either covalently bonded or forms interlacing and inseparable structures with cell walls or other similar structures; (III) when modifying agent forms aggregates from its matrix and cells; (IV) higher agglomerate organization onto surfaces; (V) internalization of modification agent.

Here we explored and overviewed emerging technologies and methodologies for enhanced performance of MFC by introduction of a modification agent into cells themselves or covering them. Summarizing these technologies, they can be classified as cell surface engineering, internalization, or artificial biofilm film formation (Figure 6). The most promising cell modification technologies involve polymeric coating formation and the internalization of living cells. While there is clear evidence of such modification-based impact on charge transfer [119], there still are some drawbacks. As some modifications are quite complex, their application in ‘real life’ MFCs could be troublesome. Main drawbacks circles around microorganism viability and proliferation as newly formed cells in MFC either should inherit or undergo the modification. Overall, cell surface modifications in tandem with other listed methodologies in this review should yield synergetic effects on the output of electricity from MFCs.

## 6. Conclusions and Future Aspects

The design of whole cell biosensors requires an optimal matrix for cell immobilization ensuring good cell performance, cell leakage prevention and electron transfer enhancement. Nevertheless, the microorganism-based biosensors show rather poor specificity and slow response because charge transfer from the cell to the electrode is delayed due to natural cell barriers (membrane and cell wall) and the cells are affected by the wide variety of chemicals. But this disadvantage can be very efficiently exploited in the design of microbial biofuel cells, as the immobilized cells can use various materials as the fuel for the generation of electrical energy.

Microbial fuel cells (MFCs) are a promising emerging technology suitable for the generation of ‘green’ electricity and bioremediation as the increase of fossil fuels causes the global energy crisis and further increases attention to environmental problems. Nevertheless, the power produced in the MFCs is still rather low for practical applications, thus the need for MFC performance improvements is of great importance. The anode as well as current-generating microorganisms are two critical components of the MFC setup. The anode provides the support for microorganism attachment, meanwhile the living cells are responsible for bacteria–electrode charge transfer mechanisms. Poor performance of the anode in MFC is still a most significant obstacle for its proper applications, therefore, efficient anode modifications are supposed to increase the surface area and efficient attachment of biofilm, which subsequently increases the electrical power production of the MFC. Another obstacle to overcome is the slow electron transfer between bacterial cells and electrode. Various chemical modifications of the cell wall or membrane are applied in order to remarkably improve the electron transfer rate and thus the power density of MFCs. Therefore, materials such as carbon nanotubes, conducting polymers, metal nanoparticles and some other metal-based nanostructures have been employed for the modification of anode and/or bacteria cell wall/membrane in order to improve the efficiency of MFCs.

The biocompatibility issues of the implantable MFCs could be resolved by electrochemically coating electrodes of BFCs with conducting polymers such as PPy or PANI or mixes of conducting polymers with chitosan or hydrogels. Various characteristics of the layers formed could be easily controlled by choosing optimal chemical and electrochemical parameters for effective electrode modifications in order to prevent inflammatory reactions while in contact with body tissues.

## Figures and Tables

**Table 1:** The description of microbial biofuel cells (MFCs) anode modification method and MFC performance. Abbreviations provided below the table.

Electricigen	Anode Modification Method	Anode Material	Electron Donor	PD, mW m^-2^	Other Remarks	Ref.
*Saccharomyces cerevisiae*	Physical absorption of CNTs, followed by physical adhesionof the cells	PU	Glucose/ MB	100	After first 24 h PD reduces to 70% of the maximum, and remains constant with continuous substitution of glucose/MB for long periods.	[14]
*Shewanella loihica*	Graft-polymerization of PANI and layer by layer self-assembling of carbon nanotubes	APTES/ ITO	Sodium lactate	34.5	Maximal current density was 6.98 μA cm^−2^, 26 times higher than plain ITO electrode.	[47]
*Saccharomyces cerevisiae*	Dip-coating of PEI and seed-mediated green synthesis growth of AuNPs followed by the biofilm formation during 72 h	CF	Glucose	2771	The single chamber architecture played a role in reducing the number of chemicals and costs.	[44]
*Saccharomyces cerevisiae*	Physical adsorption of alginate film with entrapped yeast cells	CF	Glucose	-	Current density was 0.326 A m^−2^ (CF-Yeast -algae electrode) and 0.185 A m^−2^ (CF-Yeast- Neutral Red beads electrode). Operation time was 44 days.	[52]
Classes of *Gammaproteobacteria* and *Negativicutes*	MWCNTs blended with biogenic Au and evenly spreading the paste followed by the biofilm formation	CF	Sludge	178	Start-up time 6.75 days.	[56]
*Saccharomyces cerevisiae*	Dip coating of PEI, SDBS mediated chemical growth of FeMnNPs followed by the biofilm formation	CF	Glucose	5838	Controlled FeMnNP surfactant-mediated growth was performed within a single vial under ambient conditions Relationship between the surfactant-mediated FeMnNPs and yeast biofilm development was revealed.	[43]
*Saccharomyces cerevisiae*	Dip coating of PEI followed by dipcoating of one of the QS molecules (phenylethanol, tryptophol and tyrosol). Biofilm growth.	CF	Glucose	159 * 156 135	Start-up time 3 days	[57]
*Scedosporium dehoogii*	Electrochemical deposition of the biofilm	CF	APAP	6.5	A mature biofilm was obtained at day 7	[60]
*Scedosporium dehoogii*	Electrochemical deposition of the biofilm	CF	APAP Lignin	50 16	A mature biofilm was obtained at day 7. The power density in presence of lignin of 16 mW m^−^^2^ lasted 200 h.	[61]
*Saccharomyces cerevisiae*	Cross-linking and hydrophobic interaction of yeast, PEI and CNTs	CNTs	Glucose	344	Membraneless MFC. MPD was maintained to 86% of initial value even after 8 days	[49]
*Saccharomyces cerevisiae*	Drop coating of PQ and drop coating of MWCNTs	Graphite	Glucose	1.13 10^−4^	The application of MWCNTs in anode of biofuel cell increased generated power by 69 times and generated voltage by 8 times.	[7]
*Bacillus subtilis*	Self-assembly of aldrithiol monolayer, drop coating of OsRP solution, drop coating of *B. subtilis* suspension followed by dialysis membrane fixing on the gold electrode. Drop coating of OsRP solution and drop coating of *B. subtilis* suspension on the graphite electrode	Gold Graphite	Succinate	-	The maximum current density response was ~5 μA cm^−2^. Stability test showed slight decrease response in current with time and reached approximately 73% of the initial response after 6 h.	[51]

*-power densities for MFCs based on phenylethanol, tryptophol and tyrosol, respectively. APAP—acetaminophen. APTES—γ-aminopropyltriethoxysilane. CF—carbon felt. ITO—indium tin oxide. MB—methylene blue. MWCNTs—multi-walled carbon nanotubes. NPs—nanoparticles. OsRP—osmium redox polymer.PANI—polyaniline. PD—power density. PEI—polyethyleneimine. PU—polyurethane. PQ—9,10-phenanthrenequinone. QS—quorum sensing.

## Data Availability

This is review paper, data available in referred papers.
